# The Week's Work

**Published:** 1911-08-26

**Authors:** 


### THE WEEK'S WORK.

### VACANT POST OF MEDICAL OFFICER FOR LONDON.

APPLICATIONS from candidates for the vacant post of Medical Officer for the County of London can now be received by the London County Council, owing to the approaching retirement on a pension of Sir Shirley Murphy. Candidates must not be over fifty years of age and will not be allowed to take up any private business or paid appointment in addition to their public health work, which is enough to require the whole time and energies of the Medical Officer. He will be responsible for the entire organisation and administration of the Council's school medical work, which includes of course the treatment and medical inspection of school children. The usual statutory qualifications are required, and the salary will begin at £1,250 a year with any actual out-of-pocket travelling expenses. No canvassing is allowed and the latest time for receiving applications and testimonials is October 16.

### A WARNING FOR CHICHESTER INFIRMARY.

WE can hardly share the regret which Major Chauncey expressed last week that the proposed reconstruction scheme of the Chichester Infirmary is being abandoned for lack of funds. We have followed the progress of this unfortunate scheme with some care in THE HOSPITAL, and need only refer our readers to a note in Week's Work published on July 2, 1910 (page 418). There we noted the ill-judged appeal of the Bishop for no less than £20,000, the object of which was apparently to reconstruct the sanitary block and to build a new theatre. In our Report on Chichester Infirmary published in THE HOSPITAL of October 9, 1909, the buildings were described as "possibly the best specimens extant of the old H plan of construction." Careful observation showed that all that was needed was the expenditure of some £500 on new fittings and the payment of a debt of £2,000. This last the residents did pay twelve months ago, and we are glad that they have taken to heart our criticism of the appeal for £20,000 as a waste of public money, and have withheld their support from the reconstruction scheme. This has now been reduced by the carefully withdrawn support of the locality to something like reasonable proportions, and, we are also exceedingly glad to note, during the first six months of this year the infirmary has enjoyed the new experience of paying its way. The subscribers, in short, seem to have acted with model good sense, and will be much relieved to learn that the inflated plans have for the third time been pared down. Mr. Tom Harrison, the secretary, has applied for tenders in vain: they have all proved more expensive than his funds allowed him to accept. The Duke of Richmond and Gordon has done useful work in warning the committee not to build in excess of their subscriptions, and we hope such veteran committeemen as Mr. Eugene Street and Captain Gilpin, once more restored to the board of management, will continue to enforce this warning at every meeting in future.

### AN EXAMPLE OF PERSONAL SERVICE.

IT was, we believe, through his association with the Grocers' Company that the late Mr. John Hampton Hale first became a supporter of the London Hospital. His sudden death, at the advanced age of eighty-two, a few days ago has deprived the London Hospital of a governor who had done for it plenty of useful work in his time. The crown for his services was given to him in 1896, when he was made a vice-president, but he had been a governor since 1888, the year of his Mastership of the Grocers, and had worked for several years on the house committee, of which afterwards he became chairman. This, no doubt, led him to take a wide interest in the voluntary hospitals, for he must be accorded a large share of the honour in founding the Sunday Fund, to which he gave his services as chairman of the finance committee. The British Hospital for Incurables at Streatham also engaged his services as treasurer, and he was one of the governors of the Consumption Hospital at Ventnor. He made his money as a manufacturer of Portland cement, and was a member of Lloyd's.

### WHY NEW MENTAL HOSPITALS ARE NEEDED?

KINGSEAT MENTAL HOSPITAL, Aberdeen, is to have increased accommodation, if the report just issued by Mr. John Macpherson, Commissioner in Lunacy, is to be attended to. Apparently ninety of the ninety-three beds are usually occupied, and accommodation in villas has been resorted to for patients for whom there is no room. Experience has proved here again the desirability for the convenience of administration of having a ward on each side for the observation of those patients who are allowed up. Mr. Macpherson aptly points out that the apparent increase in the number of mental cases is due to the growing proportion of the old and asthenic which is sent to mental hospitals at the present time. Apparently also the number of applicants for the post of resident medical officer in mental hospitals is also increasing, as the recent advertisement for an assistant officer issued by the Kingseat Committee was answered by two lady graduates. The post, we learn, thereupon was advertised again.

### THE CARDIFF INFIRMARY REPORT.

No copy of this report is going out unaccompanied by Colonel Bruce Vaughan's appeal for the completion of the reconstruction scheme. This, we are glad to say, has been supported so enthusiastically that for the moment the subscribers and Cardiff generally perhaps have lost sight of the routine work and financial position of their institution. Last year, to sum it up, £14,810 were spent, and £14,538 received. On the whole, considering extraneous efforts, not a bad record. The deficit was due to the interest that has still to be paid on the old-time overdraft, and, we are bound to note, the income would have fallen short by £1,800

had not the interest on the money given for endowed beds in the new wing been appropriated to maintenance. The number of in-patients was 2,483, enough to show that there are plenty of people on whom the hoped for additional income of £6,000 could be spent.

### A PAYING CONVALESCENT HOME AT MARIENBAD

A CONVALESCENT home for medical practitioners was opened at Marienbad on June 28. German and Austrian doctors who are in need of a "cure" are admitted into this new establishment on payment of twenty-eight crowns, which sum covers lodging and service for four weeks. In addition, they are exempt from the cure-tax, the music-tax, and benefit by a discount of 10 per cent. on food, 50 per cent. on the price of tickets at the municipal theatre, and are allowed free entry into the reading-rooms, etc.

### SECRETARIAL PROMOTIONS AT ST. MARY'S.

MR. C. F. SPEYER, who has held for some time the post of Steward at St. Mary's Hospital, has been succeeded by Mr. Pitt, the Senior Assistant in Mr. Ryan's, the Secretary's, office. Mr. Pitt, for the present at any rate, will perform the duties of Steward in addition to those of Senior Assistant.

### HOUSE COMMITTEE AND MEDICAL STAFF AT LOGGERHEADS.

A CERTAIN amount of friction apparently has been caused between the House Committee and the medical staff of the South Infirmary, Cork, by the recent introduction of a complaints book, which appears to be aimed at securing greater discipline. It seems that indefinite charges have been brought against the South Infirmary, particularly in relation to the alleged unsatisfactory preparations for operations. To obviate this and to regain the alleged shaken confidence of the locality, this complaints book was suggested by Mr. McNamara, and accepted by the House Committee. An interesting light on its working is thrown by a letter from Dr. Henry Corby, which was read at the monthly meeting. In the course of it he said: "It seems to me that it can hardly be part of the duty of the visiting surgeons to make daily reports with regard to the conduct of the resident doctors and the numerous young ladies who come to the hospital to learn their duties as nurses. That could only lead to disunion and unpleasantness all round. Indeed, I have learned that since your last meeting an entry honestly made in this book by Dr. Horace Townsend led to a rather unpleasant interchange of views between him and some members of the House Committee, who, unfortunately for the hospital, have never realised the importance of cultivating friendly relations with the staff, which is done in kindred institutions in Cork and elsewhere. Dr. Hegarty is primarily responsible for things being right in the operation room. . . . May I suggest that you will not now discourage him by having recourse to a system of constant criticism of his actions as house surgeon, which would be as disagreeable to those making the criticisms as it must be to himself." After some discussion it was decided that the House Committee could not go back on its decision. We very much doubt, however, if the Committee has heard the last of this book.

### A BIG CHEQUE FOR KING'S COLLEGE HOSPITAL.

WE are glad to be able to announce that £10,000 has been given anonymously to the removal fund of King's College Hospital, London, whereby the move to South London will be greatly assisted. Whatever criticisms the buildings at Denmark Hill may be open to by experts in hospital construction, the move having been undertaken, it is most important that its ultimate success should be guaranteed in so far as mere money can guarantee it.

### HOSPITALS AND THE STRIKE.

ALTHOUGH in London the industrial dispute has been lulled for the present, news arriving as we go to press shows that it is too early to say that the hospitals of Liverpool and Darlington are free from the possibility of finding it difficult to secure a regular supply of food and ice. Fortunately for the Royal Infirmary, it is not the transport workers, but the tramway owners and men, who are at the present moment of writing prolonging the struggle. On the general question, however, it is interesting to note the suggestion of Lord Montagu of Beaulieu, which appeared in Wednesday's papers, that in the case of another general strike of railwaymen communication might be assisted by the use of motor road transport. This, he said, might be secured if a free passage were guaranteed by the Government, by the immediate compilation of a national register of motorists and firms willing to lend their cars in emergency. Were this done, Lord Montagu says he would "undertake to guarantee," among other things, "the supply of milk, ice, and all necessaries to all hospitals."

### THIS WEEK'S DRUG MARKET.

THE drug market has shared in the general depression caused by the industrial disturbances, and business has been exceedingly quiet since last report appeared. Price changes have been few. Chamomiles are again dearer. The price of almond oil has been slightly advanced. The value of buchu leaves is well maintained, and ergot of rye is fetching high prices. Hydrastis canadensis is still advancing in price. The position of opium is without change, and the value is well maintained.

